# Polycarboxylate Superplasticizer-Modified Graphene Oxide: Dispersion and Performance Enhancement in Cement Paste

**DOI:** 10.3390/nano15060419

**Published:** 2025-03-08

**Authors:** Haiming Zhang, Xingyu Gan, Zeyu Lu, Laibo Li, Lingchao Lu

**Affiliations:** 1 Shandong Provincial Key Laboratory of Preparation and Measurement of Building Materials, University of Jinan, Jinan 250022, China; zhmqlu@163.com (H.Z.); ganxingyu1997@163.com (X.G.); lingchao_lu@163.com (L.L.); 2 Jiangsu Key Laboratory of Construction Materials, School of Materials Science and Engineering, Southeast University, Nanjing 211189, China; 101012819@seu.edu.cn

**Keywords:** graphene oxide, polycarboxylate superplasticizer, dispersion, flexural and compressive strength

## Abstract

Graphene oxide (GO) significantly enhances cement hydration at the nanoscale; however, its tendency to complex and agglomerate with Ca^2^⁺ in cement paste remains an unresolved issue. To improve the dispersibility and enhance the reinforcing effect of GO in cement paste, polycarboxylate (PC) superplasticizer was used to disperse GO (PC@GO). This study uniquely divided PC into two parts, with one modifying GO and the other acting as a water-reducing agent, to explore the effects on GO dispersion and analyze the rheological, carbon emission, mechanical, and hydration properties of cement paste. The experimental results show that the dispersion of GO modified by PC was improved, resulting in a significant enhancement in the performance of the cement paste containing PC@GO. The flexural and compressive strength of cement paste containing PC@GO_4_ cured for 7 days increased by 23.7% and 12.6%, respectively, meanwhile, the carbon-to-strength ratio (CI) decreased by 14.8%. In addition, the hydration acceleration period shortened by 7.50%, and the water absorption and porosity of the cement paste containing PC@GO_4_ decreased by 35.2% and 45.3%, respectively. Incorporating PC@GO into cement paste significantly enhances the dispersion of GO, substantially improves its mechanical properties, and positions it as a promising solution for the development of high-performance cementitious materials.

## 1. Introduction

Concrete remains a widely utilized construction material in the field of engineering all over the world. However, it is characterized by considerable brittleness and potential safety hazards due to its poor resistance to crack initiation and propagation [1,2,3,4,5,6,7,8]. To improve the mechanical properties of hardened cement paste, the incorporation of fibers into cement-based materials is a widely recognized method among scholars [9,10,11]. However, some fibers (such as polyvinyl alcohol fiber, polypropylene fiber, basalt fiber, etc.) may cause a reduction in the compressive strength of hardened cement paste. Moreover, these fibers primarily function to mitigate the formation of microcracks at the micrometer scale, with limited effectiveness in inhibiting the initiation and progression of cracks at the nanoscale [12,13,14,15]. Therefore, to effectively address crack formation, it is essential to control the generation and development of cracks at the nanoscale.

Recently, graphene, as a two-dimensional lamellar nanomaterial with a large specific surface area and good electrical conductivity and thermal conductivity, has been widely used as a reinforcement material in cement paste [16,17,18,19,20]. Graphene oxide (GO), a derivative of graphene containing hydrophilic oxygen functional groups (e.g., -COOH, -OH), is stable in water and has attracted attention in the cement field due to its high specific surface area and abundant oxygen functional groups [21,22,23]. Additionally, GO can regulate the morphology of hydration products and control the generation and propagation of nanoscale cracks [24]. However, achieving effective dispersion of GO in cement paste remains challenging due to the presence of calcium ions and functional groups on the surface of GO, leading to aggregation and the formation of weakened regions within the cement matrix, resulting in reduced mechanical properties [15,25,26].

Addressing the issue of GO aggregation is crucial to enhancing its practical applications in cement-based materials [13]. Zhao et al. [3] employed polycarboxylate superplasticizer (PC) to disperse GO under ultrasound treatment for a certain period, resulting in a significant enhancement of the dispersibility of GO. Donkupar et al. [27]. found that the addition of GO alone and the combined addition of GO and PC in alkali-activated slag demonstrated up to 30% and 40% enhancements in mechanical strength, respectively, compared to conventional alkali-activated slag. The steric hindrance effect, along with the combination of carboxylic groups on the PC surface with Ca^2+^, reduces the probability of GO binding with calcium ions, thereby improving its uniform dispersion in cement paste [28]. However, the effect of PC on GO dispersion has been specifically addressed less in previous studies. Therefore, it is urgent to further explore the influence of the PC content on the GO dispersion.

In this study, the effect of PC on the dispersion of GO was further evaluated through UV–vis testing, and we systematically investigated the effects of PC@GOs on the rheological, carbon-to-strength ratio (CI), mechanical, and hydration properties of cement paste. To ensure consistency in the experimental variables, a portion of the PC was utilized to disperse GO, while the remaining portion acted as a water reducer, ensuring uniform total mass across the different samples. This research provides a pathway to significantly improve the mechanical performance of cement composites.

## 2. Materials and Methods

### 2.1. Materials

**Figure 1:**
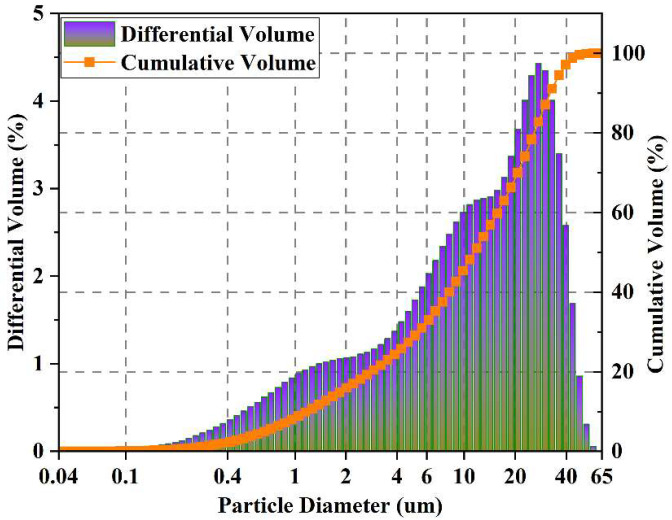
Particle size distribution of OPC.

**Figure 2:**
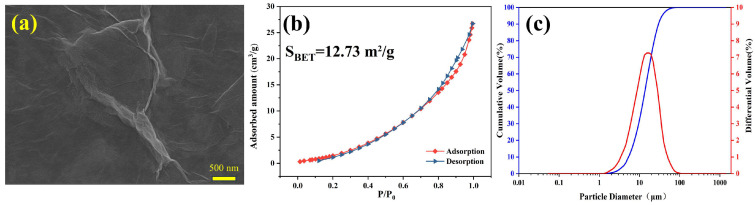
(**a**) SEM image, (**b**) N_2_ adsorption–desorption isotherm, and (**c**) particle size distribution of GO.

Ordinary Portland cement (OPC; Shandong Shanshui Cement Co., Ltd., Jinan, China) was utilized as the cementitious material. The chemical composition of the OPC was determined through X-ray fluorescence analysis, and the results are presented in Table 1. The particle size distribution obtained from a laser particle size analyzer can be found in Figure 1. The solid contents of the GO (Sixth Element Material Technology Co., Ltd., Changzhou, China) solution and PC (Jiangsu Subote New Material Co., Ltd., Nanjing, China) were 1 ± 0.2% and 30%, respectively. An SEM image, the surface area, and the particle size of the GO are shown in Figure 2.

### 2.2. Sample Preparation

#### 2.2.1. Preparation of PC@GOs and Cement Paste

To maintain a constant total amount of PC, it was divided into two parts: PC-1 and PC-2 (the total mass of PC-1 and PC-2 remained constant). PC-1 was utilized for pre-dispersing GO, while PC-2 remained constant. The GO was mixed with PC-1 at room temperature for 1 h, followed by 0.5 h of ultrasonic dispersion. Subsequently, the suspension was mixed with PC-2 and stirred for an additional 0.5 h without ultrasonication. The resulting suspension is referred to as PC@GOs and the design of the mixture is shown in Table 2. It should be noted that this experimental design is based on the research of Zhao et al. [8], where the effects of pure GO and pure PC on cement composites have been investigated.

The prepared PC@GOs was mixed with cement and water to prepare the cement paste. In accordance with the Chinese standard GB/T 1346-2011 [29], the amount of water required to achieve a standard consistency was determined to be a water–cement ratio of 0.26. The mass of PC@GOs accounted for 0.27% of the total cement. Subsequently, the cement paste was poured into a steel mold and left at room temperature for 24 h prior to demolding. After that, the samples were placed in a standard curing room maintained at 20 ± 2 °C and a humidity level of ≥95% for 3 and 7 days, respectively.

#### 2.2.2. Preparation of Simulated Pore Solution

The cement and water were added to the mixer at a water–cement ratio of 0.5 and stirred for 2 min. After curing for 20 min, the fresh cement paste was transferred into 50 mL centrifuge tubes and centrifuged at 8000 rpm for 5 min. Finally, the clear supernatant solution was collected using a membrane filter with an aperture size of 0.22 µm [30].

### 2.3. Test Methods

#### 2.3.1. Dispersion

A mixture of the pore solution and PC@GO suspension in a 1:1 ratio was prepared. Subsequently, 0.5 mL of the mixed solution was diluted with 15 mL of water, placed into a vial, and monitored for any signs of sedimentation over a period of 24 h, with observations recorded 1-, 3-, 6-, 12, and 24 h after mixing.

The modification effect of PC was characterized by monitoring changes in absorbance. The same concentration of the test solution as described above was used. The absorbance of both the upper and lower layers was measured over a 24 h period. The measurements were conducted at a wavelength of 230 nm.

#### 2.3.2. Rheological

**Figure 3:**
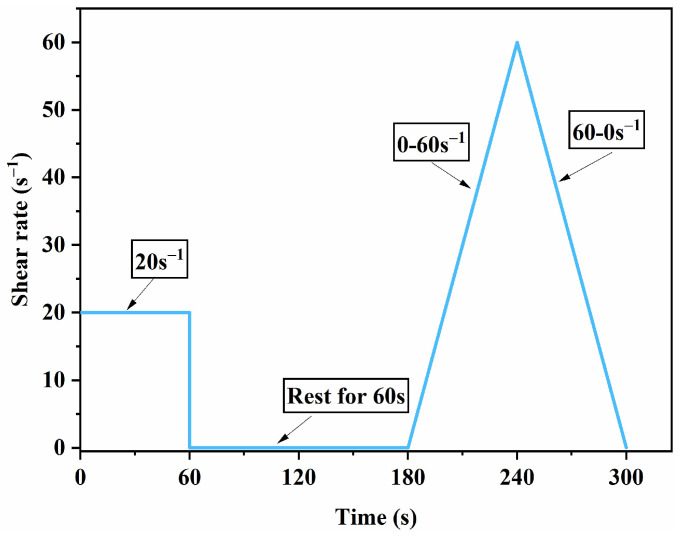
Rotary rheometer test procedure.

A rotational rheometer was employed to measure the dynamic yield stress of the cement paste. Dynamic yield stress is a basic parameter for characterizing fluidity, particularly when the microstructure is damaged, and it is closely related to the flow behavior. The testing procedures used to determine the dynamic yield stress of the cement paste consist of four steps, as shown in Figure 3.

#### 2.3.3. Mechanical Properties

The samples were subjected to compressive and flexural tests using a compressive and flexural integrated machine after 7 and 28 days of curing, respectively. The flexural strength test was conducted at a loading speed of 50 N/s, with sample dimensions of 40 mm × 40 mm × 160 mm. The compressive strength test was conducted at a loading speed of 2.4 kN/s, with sample dimensions of 40 mm × 40 mm × 40 mm. Six samples were utilized in each test.

#### 2.3.4. Carbon Emissions Evaluation

Flexural strength is a key parameter for assessing the ability of a cement-based material to resist bending stress and is one of such materials’ critical mechanical properties. In this study, the variation in the flexural strength of cement-based materials was considered in the context of evaluating CO_2_ emissions. The carbon emissions associated with cement-based materials at unit strength were calculated, which was defined as the carbon-to-strength ratio (CI) [31]. The CI was calculated as shown in Equations (1) and (2) [32].

(1) CI=E/C

(2) $$E={\rho \times V\times E}_{c}$$

where CI is the carbon intensity ratio (kgCO_2_/MPa), E is the total CO_2_ emission (kgCO_2_) of the 1 cm^3^ hardened cement paste, C is the flexural strength (MPa) of the hardened cement paste, and ρ is the density of the hardened cement paste (kg/cm^3^). V is the size of the compressive strength specimen (64 cm^3^) and E_C_ represents the CO_2_ emission generated by the production of 1 kg cement (840 × 10^−3^ kg) [33].

#### 2.3.5. Pore Structure

The pore structure of hardened cement samples was analyzed using mercury intrusion porosimetry (PoreMaster-60, American Mack Company, Shanghai, China).

#### 2.3.6. Physical Characterization

The water absorption and bulk density of cement paste were determined according to GB/T 7019-2014 [34]. 

#### 2.3.7. Heat of Hydration

The powder samples then underwent X-ray diffraction (XRD, D8 Advance, BRUKER, Shanghai, China) and simultaneous thermal analysis (TG-DSC, PE STA-8000, PERKINELMER, Shanghai, China). XRD scans were conducted using a Cu-Kα radiation source (λ = 1.5418 Å) over a 2θ range of 5° to 80° at a scan rate of 10° min^−1^ in 0.02° increments, with a tube voltage and current of 40 kV and 40 mA, respectively.

#### 2.3.8. Hydration Products

Hydration evolution tests were conducted in accordance with the Chinese standard GB/T 12959-2008 [35], utilizing a high-precision eight-channel calorimeter, the TAM Air model, manufactured by TA Instruments, New Castle, DE, USA, at a temperature of 25 °C.

## 3. Results and Discussion

### 3.1. Dispersion States and Absorbance of PC@GOs

#### 3.1.1. Dispersion States of PC@GOs

**Figure 4:**
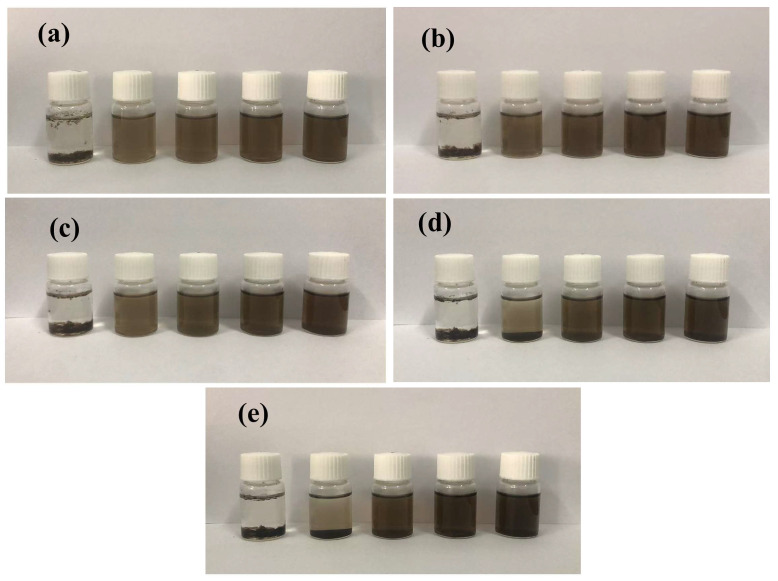
The dispersion states of PC@GOs in pore solution over time: (**a**) 1 h; (**b**) 3 h; (**c**) 6 h; (**d**) 12 h and (**e**) 24 h. In order from left to right, the samples placed in the bottles were as follows: PC@GO_0_, PC@GO_1_, PC@GO_2_, PC@GO_4_, PC@GO_8_.

The dispersion state of samples in the simulated pore solution is shown in Figure 4. As observed in [8], GO tends to agglomerate in cement pore solution due to the cross-linking effects of Ca^2^⁺. In this study, further investigation was carried out on how different ratios of PC-1 to GO could mitigate this agglomeration. PC@GO_0_ immediately agglomerated due to the complexation between -COOH groups on the GO surface and Ca^2+^ [27]. In contrast, PC@GO_1_ remained well dispersed and stable initially; however, after 12 h, a brown precipitate was observed at the bottom of the container. This was attributed to the fact that PC hinders the complexation of calcium ions with GO through the steric hindrance effect in the early stage. Additionally, the carboxyl groups on the PC surface compete with GO for the adsorption of calcium ions, thereby reducing the complexation of GO with calcium ions at the beginning of the process. The steric hindrance effect of PC and the carboxyl functional groups that induce competitive adsorption act as a protective layer for GO. Ca^2+^ slowly penetrates the PC protection and complex with the carboxyl groups on the GO surface over time. After 24 h, the dispersion of PC@GO_2_, PC@GO_4_ and PC@GO_8_ darkened slightly, but maintained good dispersion. This behavior can be attributed to the encapsulation of GO by water-reducing agent molecules, which form a double-layered electron structure and exert a steric effect, thereby reducing the likelihood of GO interacting with Ca^2^⁺ and minimizing agglomeration [36].

#### 3.1.2. Absorbance of PC@GOs

**Figure 5:**
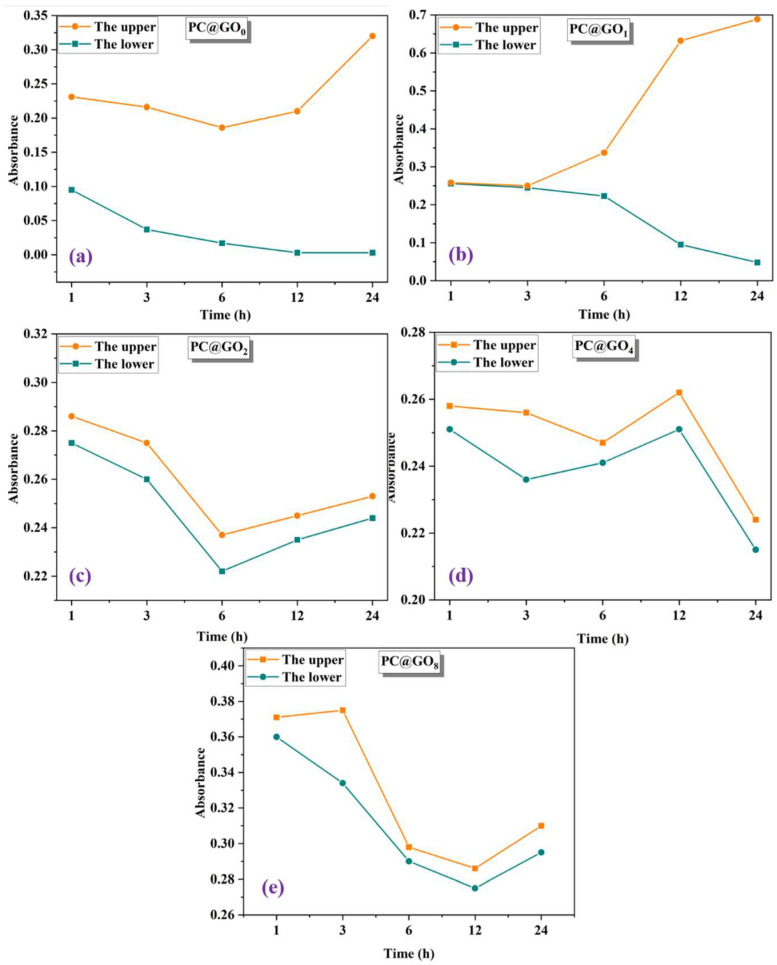
UV–vis spectrum of PC@GOs: (**a**) PC@GO_0_; (**b**) PC@GO_1_; (**c**) PC@GO_2_; (**d**) PC@GO_4_ and (**e**) PC@GO_8_.

The UV–vis absorbance of PC@GOs in simulated pore solution is illustrated in Figure 5. According to the Beer–Lambert law, the absorbance of GO is positively correlated with its dispersion [37,38], and variations in absorbance values reflect the uniformity of GO dispersion. In the simulated pore solution, GO interacts with calcium ions, leading to agglomeration and precipitation at the bottom of the quartz cuvette over time. As a result, the upper absorbance gradually increases, while the lower absorbance decreases (Figure 5b). As shown in Figure 5c, when the mass ratio of PC-1 to GO is 2:1, the difference between the absorbance of the upper-layer solution and that of the lower-layer solution begins to stabilize and shows a minimal difference. The absorbance of PC@GO_2_ sample reaches the minimum value at 6 h, which is different from that of other PC@GOs solutions after 12 h. This indicates that, at this ratio, the dispersion state of GO in the solution reaches a relatively stable level. The small difference in the concentration between the upper-layers and lower-layers implies that GO is uniformly dispersed. When the mass ratio of PC-1 to GO is 4:1 (Figure 5d), the absorbance of both the upper-layer solution and the lower-layer solution increases to a certain extent, suggesting that its stability gradually increases. The increase in absorbance may be related to the better dispersion of GO, which leads to a more uniform distribution of light-absorbing particles in the solution. When the mass ratio of PC-1 to GO is 8:1 (Figure 5e), the solution exhibits higher stability and greater absorbance. However, in this case, the amount of PC-2 used for water-reducing purposes decreases. Since PC-2 plays a crucial role in reducing the water demand of cement paste and improving its workability, this reduction may be detrimental to the cement paste. It may lead to issues such as decreased fluidity of the cement paste, which could affect the construction process and the final properties of the hardened cement-based materials. However, as the amount of PC-1 used for dispersing GO increases, more GO is encapsulated by the PC matrix, and oxygen-containing functional groups are grafted onto its surface, reducing GO’s ability to form a complex with calcium ions. This is evidenced by the minimal change in the difference between upper and lower absorbance over time. These findings demonstrate that PC effectively improves GO dispersion and reduces aggregation in simulated pore solutions.

### 3.2. Rheological Properties

**Figure 6:**
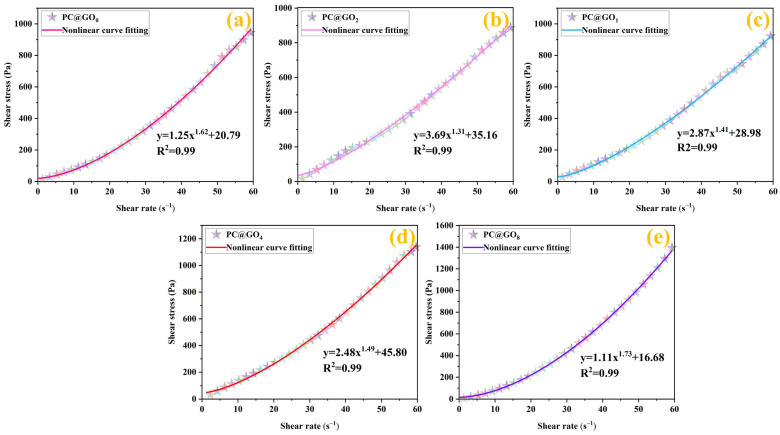
The yield stress of cement paste mixed with PC@GOs: (**a**) PC@GO_0_; (**b**) PC@GO_1_; (**c**) PC@GO_2_; (**d**) PC@GO_4_ and (**e**) PC@GO_8_.

The yield stress represents the minimum stress at which the cement pastes transitions from a solid-like to a liquid-like state [39,40,41]. Figure 6a–e represents the relationships between shear rate and shear stress for PC@GO_0_, PC@GO_1_, PC@GO_2_, PC@GO_4_, and PC@GO_8_ respectively. The dynamic yield stress of cement paste was fitted through a nonlinear curve based on the Herschel–Bulkley model, in which the intercept of the fitted curve represented the dynamic yield stress. As the ratio of PC to GO increased, the yield stress also increased, and the dynamic yield stress of PC@GO_4_ reached the largest value. The relevant equations are presented in the figures. Specifically, compared with PC@GO_0_, the dynamic yield stress of PC@GO_4_ increased by 120.30%, reaching 45.8 Pa, meaning that the flow capacity of PC@GO_4_ paste reduced. This phenomenon can be attributed to the gradual weakening of the steric hindrance and electrostatic attraction of PC with the content of PC-2, acting as a water reducer, decreased in PC@GOs. When PC excessively interacts with GO, it becomes encapsulated within the PC matrix, leading to a water-reducing effect in PC@GO_8_ and a corresponding reduction in the dynamic yield stress.

### 3.3. Mechanical Properties

**Figure 7:**
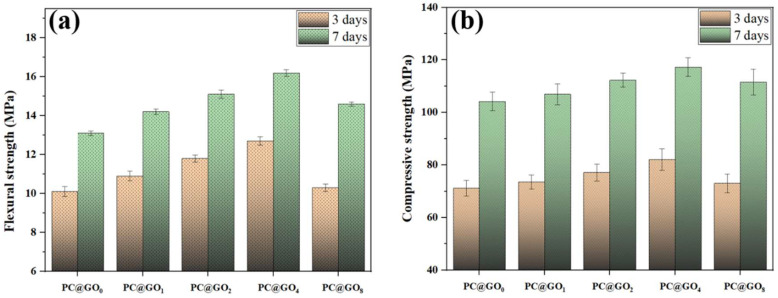
(**a**) The flexural strength and (**b**) compressive strength of cement paste mixed with PC@GOs.

Figure 7a demonstrates the effect of PC@GOs on the flexural strength of cement paste, whereas Figure 7b depicts the effect of PC@GOs on the paste’s compressive strength. As the ratio of PC-1 to GO increased, both the flexural strength and compressive strength of the cement paste cured for three and seven days initially increases, and then decreases. When the ratio of PC-1 to GO reached 4:1, the PC@GO_4_ sample exhibited significant enhancement of seven-day flexural strength and compressive strength, which were 23.7% and 12.6%, respectively, higher than PC@GO_0_. This indicates that PC effectively enhances the dispersion of GO, and the nucleation and bridging effects of GO within the cement matrix are further improved [8,42]. As the mass ratio of PC-1 to GO increases, the enhancement effect becomes more pronounced. However, it begins to weaken when the mass ratio exceeds 4:1, likely due to the overabundance of PC, which results in GO being encapsulated within the PC matrix and limits its interaction with cement particles, thereby compromising its effectiveness.

**Figure 8:**
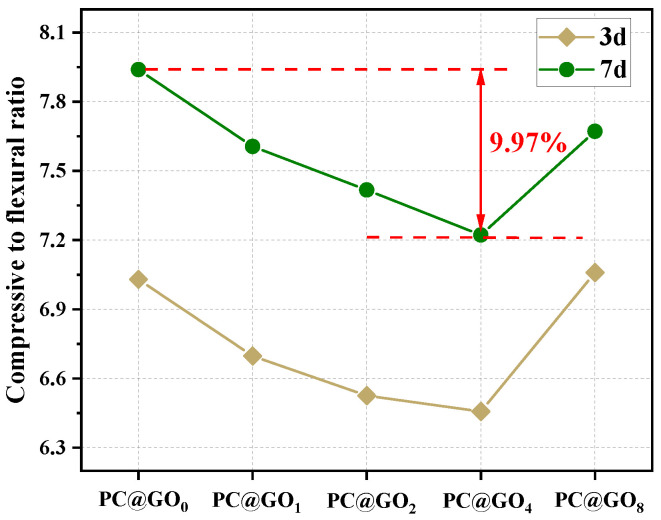
The compressive to flexural ratio of cement paste mixed with PC@GOs.

The ratios of compressive strength to flexural strength for cement paste containing PC@GOs, which are generally representative of its toughness, are shown in Figure 8. When the curing period reaches seven days, the compressive strength to flexural strength ratio of PC@GO_4_ decreases by 9.97% compared to that of PC@GO_0_, indicating a significant improvement in toughness. The modification of PC resulted in better dispersion of GO and reduced agglomeration phenomenon within cement paste, allowing GO to contribute more effectively to the bridging mechanism [42]. Furthermore, due to the two-dimensional layered structure of GO, its bridging effect was significantly more pronounced than its nucleation effect, which was manifested by the reduction of the ratios of compressive strength to flexural strength.

### 3.4. Carbon Emissions Evaluation

**Figure 9:**
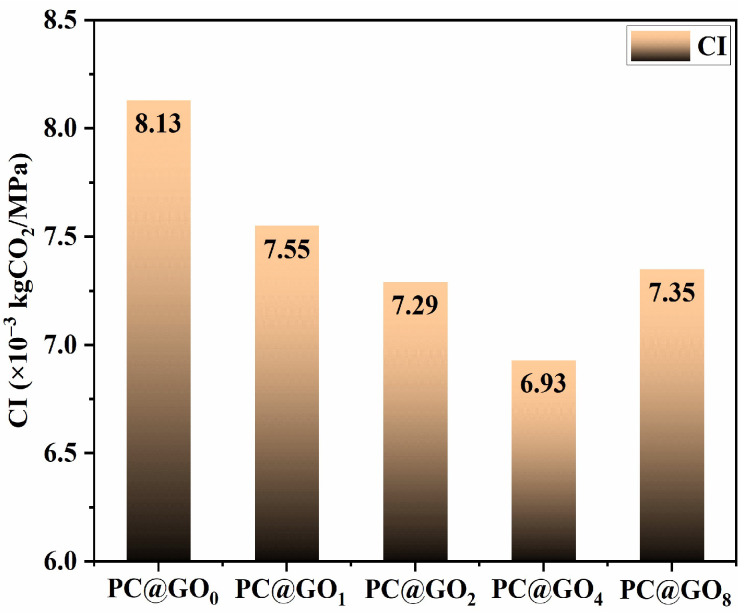
The carbon-to-strength ratio of cement-hardened paste.

As lamellar nanofibers, GO positively influenced the flexural strength of cement-based materials. Therefore, the effect of GO on the bending strength of cement-based materials was considered in this study to evaluate the carbon emissions generated by the prepared cement hardening paste. The carbon-to-strength ratio (CI), which represents the carbon emissions per unit of strength of the cement hardening paste, was used to assess the environmental impact. The effects of PC-1 to GO ratio in the cement paste on the environment were evaluated by calculating the carbon emission per unit intensity. As shown in Figure 9, the CI initially decreased, followed by a subsequent increase as the ratio of PC-1 to GO increased. When the ratio of PC-1 to GO was 4, the CI of cement-hardened paste was at its lowest, at only 6.93×10^−3^ kgCO_2_/MPa. The CI of PC@GO_4_ was 14.8% lower than that of PC@GO_0_, indicating that the introduction of PC@GOs could reduce carbon emissions while maintaining equivalent flexural strength. This is because the appropriate amount of PC led to a significant amount of GO dispersion, enhancing its nucleation and bridging effect of GO in the cement matrix.

### 3.5. Pore Structure

**Figure 10:**
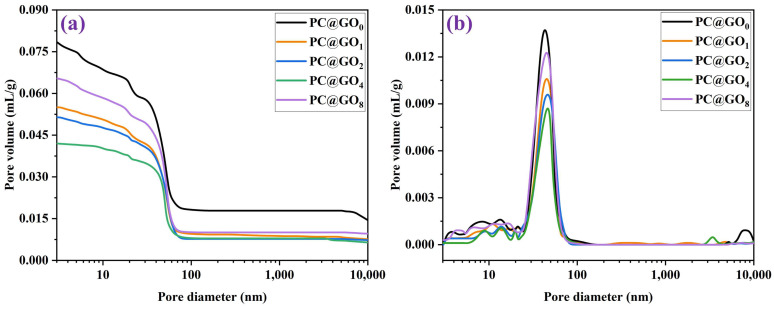
The pore structure of cement paste mixed with PC@GOs curing for seven days: (**a**) pore volume size distribution and (**b**) cumulative pore volume.

**Figure 11:**
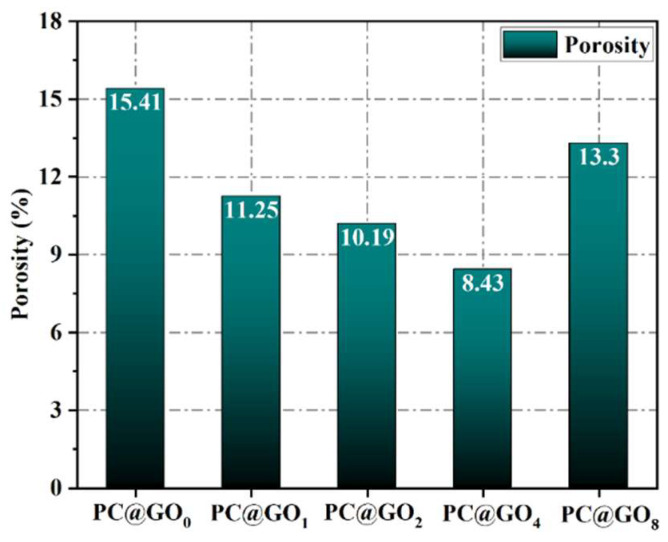
The porosity of cement pastes mixed with PC@GOs curing for seven days.

The MIP technique was employed to assess the pore structure of the cement samples and to quantify the impact of PC@GOs on refining the pore structure of the cement matrix, as illustrated in Figure 10 and Figure 11. As the ratio of PC-1 to GO increased, there was an initial increase followed by a decrease in the total porosity of the cement sample. PC@GO_4_ exhibited a total porosity of 8.43%, which was 45.3% lower than that observed for PC@GO_0_. The decrease in porosity and the refinement of pore structure observed in PC@GO_4_ contributed to improved mechanical strength and durability [8]. The enhanced dispersibility of GO facilitates the creation of more nucleation sites, promoting the formation of cement hydration products and consequently reducing porosity. Moreover, this decrease in porosity is directly related to the increased compressive strength and reduced water absorption of the cement paste, providing further evidence of the role of PC@GOs in improving the properties of cement paste.

### 3.6. Physical Characterization

**Figure 12:**
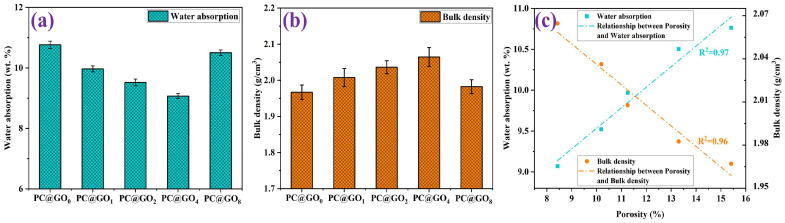
(**a**) Water absorption, (**b**) bulk density and (**c**) the relationship between porosity, water absorption and bulk density of cement hardened paste mixed with PC@GOs curing for seven days.

Figure 12a illustrates the water absorption of the PC@GOs sample, highlighting a trend of decreasing and then increasing water absorption as the ratio of PC-1 to GO increases. The lowest amount of water absorption (9.07%) occurred at a PC-1 to GO ratio of 4:1, which was 15.7% lower than that of PC@GO_0_. This can be attributed to the excellent dispersion effect of PC-1 on GO, leading to an increase in hydration products and a denser microstructure. The lower water absorption observed in the cement paste further confirms this improvement. Furthermore, the fitting curve of water absorption and porosity for cement paste mixed with PC@GOs after curing for seven days was illustrated in Figure 12c. A stronger linear relationship (R^2^ = 0.97) between water absorption and porosity was established, demonstrating the functional interdependence of these two properties [43]. The significant correlation further supports the effectiveness of PC@GOs in improving the performance of cement paste by reducing porosity and water absorption, and consequently enhancing the durability and strength [43].

The influence of the PC-1 to GO ratio on the bulk density of cement paste is shown in Figure 12b. The maximum bulk density of 2.06 g/cm^3^ was achieved at a PC-1 to GO ratio of 4:1. Compared with PC@GO_0_ and PC@GO_8_, the bulk density of PC@GO_4_ increased by 4.5% and 4%, respectively. The bulk density increased proportionally, with a strong linear correlation between these two properties (R^2^ = 0.96). This finding further emphasizes that the excellent dispersion effect of PC-1 on GO contributes to a denser microstructure in the cement paste, which enhances the durability of the cement paste, highlighting the positive impact of PC@GOs on the performance.

### 3.7. Hydration Evolution

**Figure 13:**
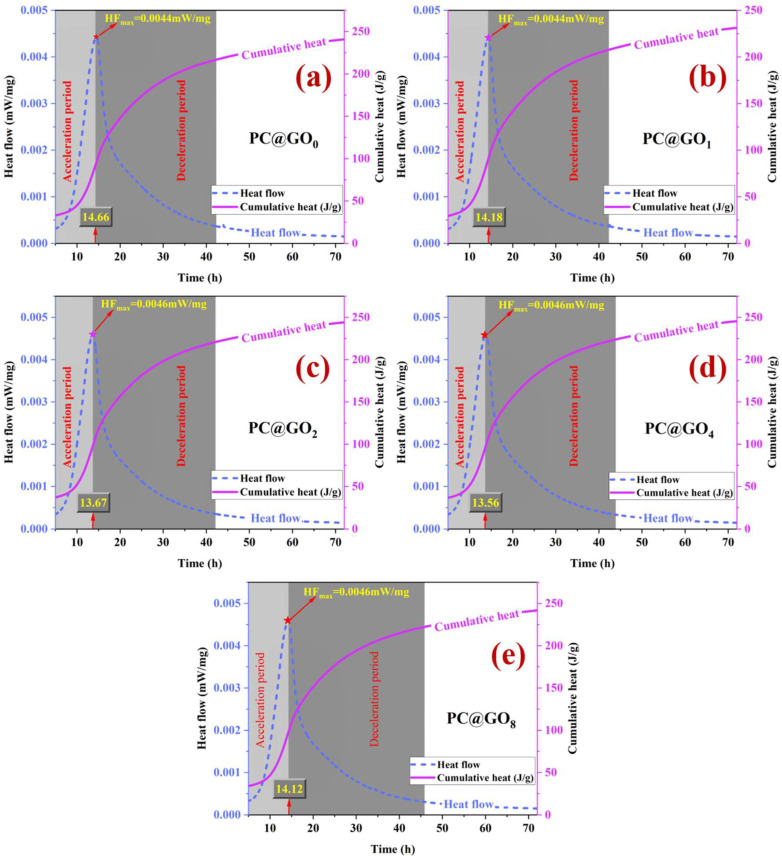
Hydration heat evolution of cement paste mixed with PC@GOs: (**a**) PC@GO_0_; (**b**) PC@GO_1_; (**c**) PC@GO_2_; (**d**) PC@GO_4_ and (**e**) PC@GO_8_.

The thermal evolution of hydration in PC@GOs within 72 h is shown in Figure 13. As the ratio of PC-1 increased, the time required for the modified cement paste to reach the maximum heat flow was shortened, indicating that PC@GOs effectively reduce the acceleration period of cement hydration. Specifically, compared with PC@GO_0_, the hydration acceleration period of PC@GO_4_ in cement paste was shortened by 7.50%, reaching just 13.56 h. This is attributed to the fact that PC-1 prevents GO aggregation through weak electrostatic repulsion and poor hydrophilicity, resulting in a steric effect that increases the availability of nucleation sites for cement hydration and thereby accelerating the hydration process [44]. However, the maximum heat flow time of PC@GO_8_ modified cement paste is longer than that of PC@GO_4_. This delay in the heat release peak may be due to an excessive amount of PC-1 encapsulating GO, which reduces the number of nucleation sites for cement hydration and ultimately slows down the overall hydration process [45,46].

### 3.8. Hydration Products

**Figure 14:**
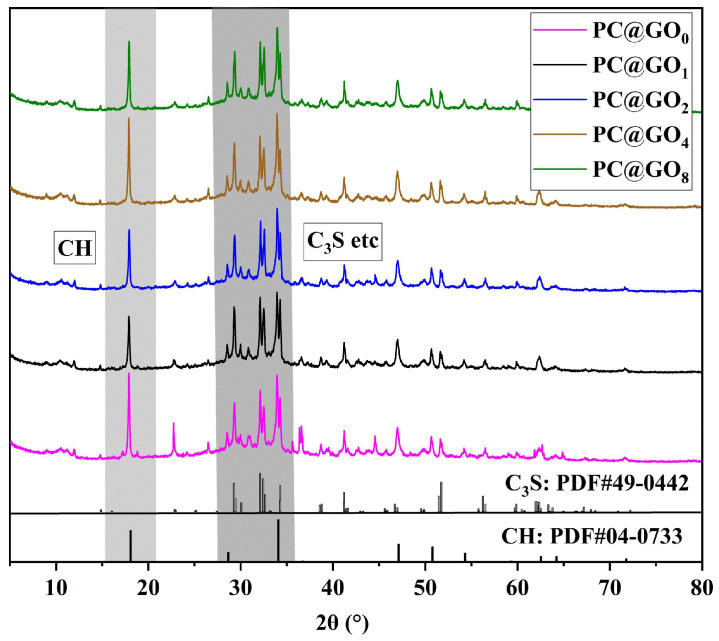
XRD pattern of cement hydration products.

The XRD pattern of cement hydration products is presented in Figure 14, which reveals the presence of a typical hydration product associated with Portland cement. Due to the relatively low dosage of PC@GOs, no discernible diffraction patterns corresponding to PC and GO can be observed. The quantity of early CH, a byproduct of cement hydration, serves as an indicator of both the amount of C-S-H produced and the extent of cement hydration. A higher peak intensity of the CH peak typically corresponds to a more advanced degree of hydration. The cement paste modified with PC@GO_4_ exhibits the most prominent CH peak at 18.3°, suggesting that a PC-1 to GO ratio of 4:1 optimizes the hydration promotion and dispersion effects of GO. Based on Figure 14, it can be concluded that the incorporation of PC@GOs influences the overall quantity of hydration products, but does not result in the formation of new crystalline phases during the cement hydration process.

## 4. Conclusions

PC@GOs were utilized to reinforce cement composites. The combined effects of PC-1 on GO dispersion and PC-2 on water reduction in cement paste were investigated at the same PC content to evaluate the enhancement effect of PC@GOs on rheological, carbon emissions, mechanical, and hydration properties of Portland cement. The results are as follows:

(1) PC@GOs can be uniformly dispersed in the simulated alkaline pore solutions of cement paste.
(2) PC@GOs can effectively shorten the acceleration period of cement hydration. Compared with the blank group, the hydration acceleration period of PC@GO_4_ cement paste was shortened by 7.50%, to as little as 13.56 h.
(3) The mechanical properties of PC@GO_4_ cement paste were significantly improved. Compared with the blank group, after seven days, the flexural strength and compressive strength of the PC@GO_4_ sample increased by 23.7% and 12.6%, respectively.
(4) PC@GO_4_ had the lowest water absorption rate and total porosity, which were 9.07% and 8.43%, respectively. Compared with the blank group, the water absorption rate and total porosity of PC@GO_4_ were decreased by 35.2% and 45.3%, respectively.
(5) The incorporation of PC@GOs had a significant effect on the carbon emissions per unit strength of cement hardening paste. The CI of PC@GO_4_ was reduced by 14.8% compared with PC@GO_0_.

## Figures and Tables

**Table 1:** Chemical compositions of OPC.

Compositions	CaO	SiO_2_	Al_2_O_3_	Fe_2_O_3_	SO_3_	MgO	K_2_O	Na_2_O	LOI
Content	67.70	16.29	5.31	3.58	2.78	2.56	0.50	0.29	0.99

**Table 2:** Mix design of PC@GOs (the subscript in PC@GOs denotes the ratio of PC-1 to GO).

Sample	PC-1: GO Mass Ratio	PC-1 (g)	PC-2 (g)	GO (g)	Cement (g)	Water (g)
PC@GO_0_	0:1	0	1.44	0.18	600	156
PC@GO_1_	1:1	0.18	1.26	0.18	600	156
PC@GO_2_	2:1	0.36	1.08	0.18	600	156
PC@GO_4_	4:1	0.72	0.72	0.18	600	156
PC@GO_8_	8:1	1.44	0	0.18	600	156

## Data Availability

Dataset available on request from the authors.
